# Keystone Veterinary Medical Association

**Published:** 1896-09

**Authors:** W. L. Rhoads

**Affiliations:** Secretary


Secretary.

### KEYSTONE VETERINARY MEDICAL ASSOCIATION.

This Association resumed its regular monthly winter meetings at the hall northwest corner of Broad and Filbert Streets, on Tuesday evening, September 8, 1896. President Hart in the chair. Secretary Rhoads marked the members present. Report of progress was received from the Committee on Certificates, and that all suggestions had been harmoniously decided upon and the certificates would be ready for delivery at the October meeting.

After listening to the most gratifying reports from members Hart and Hoskins of the Convention at Buffalo, with a brief summary of programme and recital of the great hospitality enjoyed, it was with keen regret that many voiced their disappointment at not having been able to go.

A full winter's work was planned for, and the October meeting will be favored with a paper by Dr. W. Horace Hoskins on "Some Aspects of Association Work in the Future." The first paper in the series of half-hour talks, a plan inaugurated by the Association for the entertainment of the members during the coming season, will be by Dr. J. Cheston Morris, member of the Devon Cattle Club, on some thoughts and statistics in dairy-products, personal experiences in the proper preparation of milk for sale in the markets, and the value of separator products of milk.

A number of cases were reported, and an anatomical freak was reported by Dr. Hoskins in a male Angora cat, recently castrated and placed in his sanitarium for completion of recovery subsequent to operation. Subject about ten months old; local parts little disturbed by operation; animal extremely anæmic, with complete anorexia, during a period of two weeks eating nothing but a few spoonfuls of milk, refusing every other kind of food offered. The animal was listless, given to lying on the broad side a great deal, right side invariably; a fetid diarrhœa preceded death a short time, the animal dying two weeks after admittance. Diagnosis: Pernicious anæmia. Treatment: All kinds of food, milk, eggs, fish, meats (cooked and uncooked), tonics, pepsin, iron, and nux vomica. Autopsy: Heart in abnormal position resting against right thoracic wall; remnants of right lung only; left lung divided in five lobes, firmly attached to spinal column; each lobe divided in its entirety. Partially developed diaphragm. Large omentum, estimated to be increased in size one-half, very thick, and imbedded with small waxy-like deposits, somewhat resembling miliary tuberculosis; omentum extended to the thoracic cavity and appeared to aid in sustaining the heart in place of pleural folds of mediastinum. Liver displaced, and very much like a horse-chestnut in shape, very dark in color, and rough on exterior, situated almost entirely on right side of abdomen and resting against internal abdominal wall. All other organs very pale and waxy in color, but otherwise normal in appearance.

Delegates were appointed to the Pennsylvania State meeting at Reading on October 8, 1896.

W. L. Rhoads, D.V.S.,
Secretary.

